# Challenges of Ascertaining National Trends in the Incidence of Coronary Heart Disease in the United States

**DOI:** 10.1161/JAHA.114.001097

**Published:** 2014-12-03

**Authors:** Earl S. Ford, Véronique L. Roger, Shannon M. Dunlay, Alan S. Go, Wayne D. Rosamond

**Affiliations:** Division of Population Health, National Center for Chronic Disease Prevention and Health Promotion, Centers for Disease Control and Prevention, Atlanta, GA (E.S.F.); Division of Cardiovascular Diseases, Department of Internal Medicine, Mayo Clinic, RochesterMN (R., S.M.D.); Division of Research, Kaiser Permanente Northern California, Oakland, CA (A.S.G.); Departments of Epidemiology, Biostatistics and Medicine, University of California, San Francisco, CA (A.S.G.); Department of Health Research and Policy, Stanford University School of Medicine, Palo Alto, CA (A.S.G.); Department of Epidemiology, Gillings School of Global Public Health, University of North Carolina, Chapel Hill, NC (W.D.R.)

**Keywords:** coronary heart disease, incidence, trends

## Introduction

Despite major therapeutic advances, the public health burden associated with coronary heart disease (CHD) remains enormous with approximately 525 000 people predicted to have a new myocardial infarction (MI) in 2013, ≈15.4 million estimated to be living with CHD in 2013, and ≈1 346 000 people hospitalized in 2009 for CHD.^1^

There are a variety of ways to measure the population impact of a disease including prevalence, associated morbidity and mortality, quality of life, health care utilization, and economic costs, and one of the most critical is disease incidence. From a surveillance perspective in the United States, the national vital statistics data system provides information about the death rate for CHD, various national data systems provide estimates of hospitalizations for CHD and outpatient visits for CHD, and national data systems provide data about levels of risk factors for CHD. The data systems allowing for estimates of prevalent CHD are less robust as they rely primarily on self‐reported information.

A particularly glaring gap in our knowledge base has been the lack of nationally representative data to measure the incidence of CHD. Measuring incidence of a disease is particularly salient because incidence (1) is a key measure in helping to define the burden of a disease and identify high‐risk populations, (2) provides valuable information in helping decision makers set public health priorities, and (3) is a more relevant measure to assess the collective influence of risk factors in a population than prevalence. Consequently, tracking incidence of a disease in populations can: (1) yield timely data about potentially unfavorable changes in incidence that may prompt a search for explanations and corrective actions to redirect the course of a disease in a population, (2) provide valuable feedback in assessing efforts to control a disease, and (3) generate useful information for updating priorities regarding health promotion and disease prevention. The reasons why a national surveillance system to track CHD incidence in the United States has never been developed are not entirely clear but may relate to the cost and complexity of implementing such a system.

Our objective is to review the fragmented data that may have bearing on incidence of CHD in the United States. Because national data about incident CHD are not readily available, we will examine various facets of CHD epidemiology—including mortality, hospitalizations and case‐fatality, prevalence, risk factors, and predicted risk—that may provide insights about national trends in the incidence of CHD. Incidence, prevalence, and mortality are interrelated,^2–3^ and, hence, we will explore data for the latter two important population surveillance parameters. Declining mortality rates have been postulated as possible evidence for declining incidence rates, and, therefore, we examine published trends in mortality as well as in case‐fatality rates that have bearing on overall mortality rates from CHD. Furthermore, trends in hospitalizations for MI have often been used as a surrogate measure for trends in incidence of this condition, and consequently, we review national and regional data on this topic. Because the sum total of risk factors for CHD drive the incidence of this disease, we assess trends in individual risk factors as well as predicted risk calculated from major CHD risk factors. Finally, we review regional data about trends in CHD incidence from community surveillance and cohort studies.

## Mortality

**Figure 1.**
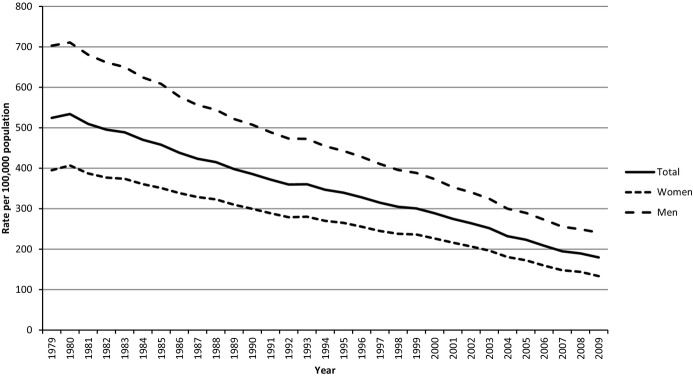
Age‐adjusted mortality rates from CHD for adults aged ≥25 years, United States. Results were generated with WONDER using the Compressed Mortality File of the National Vital Statistics System. For the period 1979–1999, International Classification of Diseases 9 codes 410‐414 and 429.2 were used. For 2000–2009, International Classification of Diseases codes I20‐O25 were used. Results were age‐adjusted to the projected year 2000 US population. CHD indicates coronary heart disease.

**Figure 2.**
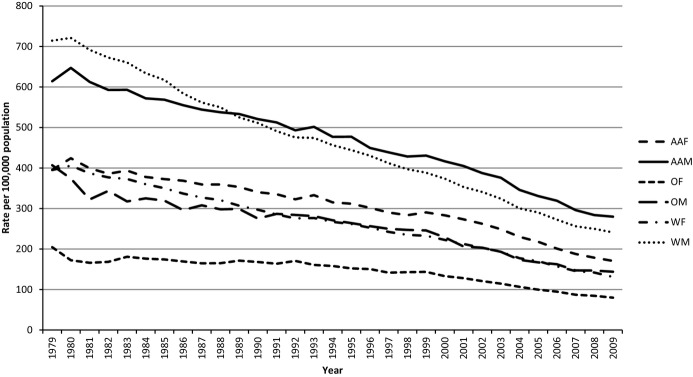
Age‐adjusted mortality rates from CHD for adults aged ≥25 years, by race and gender, United States. Results were generated with WONDER using the Compressed Mortality File of the National Vital Statistics System. For the period 1979–1999, International Classification of Diseases 9 codes 410‐414 and 429.2 were used. For 2000–2009, International Classification of Diseases codes I20‐O25 were used. Results were age‐adjusted to the projected year 2000 US population. AAF indicates African‐American females; AAM, African‐American males; CHD, coronary heart disease; OF, other females; OM, other males; WF, white females; WM, white men.

The category “diseases of the heart” has long been and continues to be the leading cause of death in the United States based on data from death certificates.^4^ After increasing during the first part of the 20th century, the mortality rate attributed to CHD peaked during the late 1960s and reversed course starting a prolonged and continuing decline.^5–6^ From 1980 through 2009, age‐adjusted CHD mortality has decreased by 66% among men and 67% among women (Figure 1). Furthermore, age‐adjusted rates decreased by 60% among African American women, 57% among African American men, 68% among white women, and 67% among white men (Figure 2). CHD mortality was defined as International Classification of Diseases (ICD)‐9 codes 410‐414 and 429.2 or ICD‐10 codes I20‐I25. Regional studies such as the Framingham Heart Study, the Minnesota Heart Survey, Honolulu Heart Program, and the Atherosclerosis Risk in Communities Study (ARIC) also described declining rates of CHD mortality.^7–11^ The factors contributing to the decline have been debated, and a combination of treatment and improvements in population levels of risk factors for CHD has been credited with lowering the CHD mortality rate.^12–17^ The declining mortality rates raised the prospect of declining incidence rates. Because mortality rates are subject to a number of influences such as disease severity, case fatality, changes in risk factors, improved treatment, and incident or new cases,^18^ declining mortality rates alone cannot automatically be equated with declining incidence rates.

## Hospitalizations

Several large data sets have provided information about trends in hospitalizations for MI (Table 1).

**Table 1.** Large Studies of Trends in Hospitalization Rates for Myocardial Infarction in the United States

Reference	Data Source	Study Period	Change in Rates (Per 100 000)	Discharge Diagnosis	Validation of Discharge Diagnoses
Nallamothu^19^	Acute Care Tracker Database	2002–2005	309 to 266	Principal	No
Fang^20^	National Hospital Discharge Survey	1979–1981 to 1985–1987	215 to 342	Principal	No
		1985–1987 to 2003–2005	342 to 242		
Chen^21^	Medicare fee‐for‐service beneficiaries	2002–2007	1131 to 866	Principal	No
Wang^22^	National inpatient sample	2001–2007	314 to 222	Principal	No

Based on the Acute Care Tracker data base, a proprietary administrative database that included 458 US hospitals, rates of hospitalization for MI based on principal diagnosis ICD‐9 codes decreased from 309 in 2002 to 266 per 100 000 population in 2005.^19^ The numbers of total discharges and coronary revascularizations compared reasonably well with estimates from the National Hospital Discharge Survey, but the diagnoses of MI were not specifically validated. An analysis of data from the National Hospital Discharge Survey showed that the rate of hospitalizations for MI using the first‐listed diagnosis code increased from 215 in 1979–‐1981 to 342 per 100 000 population in 1985–‐1987, remained relatively level until 1996, and then declined to 242 per 100 000 population in 2003–‐2005.^20^ No validation of discharge diagnoses was done. The rate of hospitalizations for MI using principal diagnosis codes among Medicare fee‐for‐service beneficiaries dropped from 1131 in 2002 to 866 per 100 000 person‐years in 2007.^21^ Discharge diagnoses were not validated. An analysis of data from the National Inpatient Sample of the Healthcare Cost and Utilization Project from 2001 to 2007 found that the rate of hospitalization from MI based on the principal diagnosis dropped from 314 to 222 per 100 000 population, and decreases were observed in most demographic subgroups.^22^ The validity of the discharge diagnoses over time remained untested in this data set. However, these studies were not able to identify incident CHD or to examine the impact of changes in diagnostic criteria for MI on hospitalization rates. Furthermore, validation of hospitalizations for MI diagnostic codes has generally not been done in these studies.

## Case‐Fatality

Several measures of case‐fatality rates can be conceptualized in terms of time frame: in‐hospital mortality, 28‐ or 30‐day mortality, and 1‐, 2‐, 3‐, and 5‐year mortality (Table 2).

**Table 2.** Selected Studies of Changes in Case‐Fatality Rates for Hospitalizations for Myocardial Infarction or Incident Coronary Heart Disease in the United States

Reference	Study	CHD Event	Period	Changes in Case‐Fatality Rate (%)							
				In‐Hospital	28‐Day	30‐Day	3‐Months	1‐Year	2‐Year	3‐Year	5‐Year
Elveback^7^	Rochester, MN	Incident MI	1965–1969 to 1970–1975			18.0 to 9.3					40.0 to 34.0
Gillum^23^	Minnesota Heart Survey	Any MI	1970–1980	Men: 16.7 to 11.9							
				Women: 16.6 to 12.2							
Pell^24^	Du Pont Company	Incident MI	1957–1959			30.4					
			1972–1974			34.8					
			1981–1983			24.3					
Goldberg^25^	Worcester Heart Attack Study	Any MI hospitalization	1975–1984	22.2 to 15.1							
		Incident MI	1975–1984	20.1 to 12.6							
Reed^9^	Honolulu Heart Program	Incident CHD	1966–1985			↑					↑
Keil^26^	Pee Dee, SC	Any MI	1978–1985	Total: 14 to 9.9							
				WM: 12.3 to 7.4							
				WW: 20.0 to 7.0							
				BM: 17.7 to 17.4							
				BW: 9.1 to 29.4							
McGovern^18^	Minnesota Heart Survey	Incident MI hospitalization	1985–1990		Men: 13 to 10					Men: 21 to 18	
					Women: 15 to 12					Women: 29 to 24	
Rosamond^11^	Atherosclerosis Risk in Communities Study	Any MI hospitalization	1987–1994		Men: 4.1%/y ↓						
					BM: 2% ↑						
					WM: 5.1% ↓						
					Women: 9.8%/y ↓						
					BW: 3.1% ↓						
					WW: 12.1% ↓						
Goldberg^27^	Worcester Heart Attack Study	Incident MI hospitalization	1975–1978	17.8				12.0*	17.0*		31.0
			1981–1984	14.9				13.0*	19.0*		32.0
			1986–1988	17.0				10.0*	16.0*		29.0
			1990–1991	13.2				13.0*	19.0*		31.0
			1993–1995	11.7				11.0*	17.0*		―
Ergin^28^	National Health and Nutrition Examination Survey I EFS	Incident CHD	1971–1982		Total: 23.4; WM: 27.2; WW: 15.3; BM: 39.2; BW: 26.5						
			1982–1992		Total: 16.6; WM: 19.0; WW: 14.2; BM: 10.8; BW: 16.6						
Peterson^29^	National Registry of Myocardial Infarction	Any MI hospitalization	1990–2006	10.4 to 6.3							
		Any STEMI		11.5 to 8.0							
		Any NSTEMI		7.1 to 5.2							
Wellenius^30^	Medicare beneficiaries	Any MI hospitalization	1984–2003	WM: 22.7 to 10.1		WM: 25.2 to 14.3		WM: 40.3 to 28.7			
				WW: 23.1 to 10.3		WW: 25.2 to 14.4		WW: 39.3 to 28.8			
				BM: 18.6 to 11.2		BM: 20.9 to 15.5		BM: 37.2 to 34.8			
				BW: 20.0 to 11.2		BW: 21.6 to 15.1		BW: 38.5 to 33.8			
Parikh^31^	Framingham Heart Study, Framingham Heart Study Offspring	Incident MI	1960–1969 to 1990–1999			73.0 ↓		65.0 ↓			64.0 ↓
		Incident MI‐ECG				62.0 ↓		58.0 ↓			64.0 ↓
		Incident MI‐marker				78.0 ↓		69.0 ↓			55.0 ↓
Floyd^32^	Worcester Heart Attack Study	Incident MI hospitalization	1975–2005	19.5 to 9.5							
Fang^20^	National Hospital Discharge Survey	Any MI hospitalization	1979–1981 to 2003–2005	17.8 to 8							
Yeh^33^	Kaiser Permanente Northern California	Incident MI hospitalization	1999–2008			10.5 to 7.8					
		Incident NSTEMI hospitalization				10.0 to 7.6					
Roger^34^	Olmsted County, MN	Incident MI hospitalization	1987–2006			−4.3%/y					
McManus^35^	Worcester Heart Attack Study	Any STEMI hospitalization	1997	11.1		13.2		10.6*			
			1999	9.9		13.0		14.0*			
			2001	13.5		15.8		15.4*			
			2003	8.4		10.0		8.3*			
			2005	9.7		11.4		8.4*			
		Any NSTEMI hospitalization	1997	12.9		16.0		23.1*			
			1999	13.1		17.0		27.6*			
			2001	10.9		16.5		26.1*			
			2003	8.9		13.7		25.6*			
			2005	9.5		14.0		18.7*			
Nguyen^36^	Worcester Heart Attack Study	Any MI hospitalization	1986–1988 to 2003–2005	Men, <65 years: 7.1 to 2.2 Women, <65 years: 9.6 to 5.3							
				Men, age 65 to 74 years: 14.3 to 8.2 Women, age 65 to 74 years: 19.6 to 11.5							
				Men ≥75 years: 30.2 to 13.3 Women ≥75 years: 29.6 to 12.6							
Coles^37^	Worcester Heart Attack Study	Incident MI	2001–2007				11.1 to 7.9*	17.1 to 12.7*	25.6 to 18.6*		
Rosamond^38^	Atherosclerosis Risk in Communities Study	Incident MI hospitalizations	1987–2008		Men: 3.4%/y ↓, WM: 3.5%/y ↓, BM: 3.4% ↓, Women: 2.9% ↓, WW: 3.0%/y ↓, BW: 2.6% ↓						

BM indicates black men; BW, black women; CHD, coronary heart disease; ECG, electrocardiogram; NSTEMI, non‐ST‐segment myocardial infarction; STEMI, ST‐segment elevation myocardial infarction; WM, white men; WW, white women.

* Post‐discharge.

Numerous publications have documented improvements in the in‐hospital or short‐term case‐fatality rate.^7,11,18,20,23–26,23–34,23–39^ The first indications that CHD case‐fatality rates had improved emerged during the 1960s.^7^ Since then, case‐fatality rates have generally improved steadily. Fewer data are available concerning the long‐term survival of people who develop CHD. In Rochester, MN, the 5‐year mortality rate from 1965–1969 to 1970–1975 decreased from 40.0% to 34.0%.^7^ An early report from the Worcester Heart Attack Study failed to observe improved post‐discharge long‐term survival in patients who sustained an MI in 1975, 1978, or 1981.^40^ A subsequent analysis of data from this study again failed to find improvements in 1‐, 2‐, and 5‐year survival rates for patients who were discharged during 1975–1978, 1981–1984, 1986–1988, and 1990–1991.^27^ More recently, 1‐year survival for patients discharged with an ST‐segment elevation MI (STEMI) during 2003 and 2005 and for patients discharged with non‐ST‐segment MI (NSTEMI) during 2005 improved,^35^ and 1‐ and 2‐year mortality rates from 2001 to 2006 decreased from 17.1% to 12.7% and 25.6% to 18.6%, respectively.^37^ In the Minnesota Heart Survey, 3‐year mortality after hospitalization for MI decreased from 21% in 1985 to 18% in 1990 among men and from 29% to 24% among women.^18^ Among Medicare beneficiaries, 1‐year mortality after a MI decreased from 40.2% in 1984 to 34.5% in 2003.^30^ In the Framingham Study, 1‐ and 5‐year mortality among adults who had an MI decreased by 65% and 64%, respectively, during the period from 1960 to 1999.^31^

## Prevalence

Broadly speaking, prevalence represents the net sum of input (incidence) and outflow (mortality). Thus, information about trends in CHD prevalence may shed light on the incidence of CHD. Information about the prevalence of CHD comes from national surveys, cohort studies, and regional surveillance systems. National surveys like the National Health and Nutrition Examination Survey (NHANES), National Health Interview Survey (NHIS), and Behavioral Risk Factor Surveillance System (BRFSS) use questionnaires to collect data to estimate the prevalence of CHD. Because these systems rely on self‐reported information, such information is particularly susceptible to various biases.

Several analyses of NHANES data have been undertaken. Among NHANES participants aged 40 to 74 years, estimates of the prevalence of self‐reported MI were 6.3% during 1971–1975, 5.6% during 1976–1980, and 5.7% during 1988–1994.^41^ Among adults aged 35 to 54 years who participated in NHANES, the prevalence of self‐reported MI was 2.5% during 1988–1994 and 2.2% during 1999–2004 among men and 0.7% during 1988–1994 and 1.0% during 1999–2004 among women.^42^ Analysis of NHANES data by the National Heart, Lung, and Blood Institute showed that the prevalence of self‐reported MI has declined from 1971–1975 to 2005–2008 most clearly among whites and among men.^6^

To examine the recent trend in CHD prevalence, we used NHANES data of adults aged ≥20 years from 1999 to 2012 (Table 3).^43^ CHD was defined as ever having been told by a doctor or other health professional that the participant had CHD, angina pectoris, or a heart attack. The unadjusted prevalence showed little change during the 10‐year period. After adjustment for age, the prevalence of CHD increased from 6.3% during 1999–2000 to 6.9% during 2003–2004 and then decreased to 5.2% during 2009–2012, and the overall trend showed a decrease (*P* for linear trend=0.001). Furthermore, decreases in the age‐adjusted prevalence of self‐reported CHD were noted for men, women, whites, African Americans, adults who had not completed high school or with education beyond high school, adults without diagnosed diabetes, and adults with a body mass index <30 kg/m^2^.

**Table 3.** Unadjusted and Age‐Adjusted Prevalence (%, SE) of Self‐Reported CHD Among Adults Aged ≥20 Years, National Health and Nutrition Examination Survey 1999–2012

	1999–2000	2001–2002	2003–2004	2005–2006	2007–2008	2009–2010	2011–2012	*P* Linear Trend
Unadjusted results								
Total	5.8 (0.4)	5.9 (0.5)	6.8 (0.8)	6.1 (0.5)	5.6 (0.3)	5.5 (0.4)	5.4 (0.4)	0.165
Age, y								
20 to 44	1.0 (0.3)	1.0 (0.2)	0.7 (0.3)	1.1 (0.3)	0.8 (0.2)	1.0 (0.3)	1.0 (0.3)	0.996
45 to 54	4.9 (0.9)	3.7 (0.8)	4.3 (0.8)	4.2 (0.6)	3.7 (0.6)	4.0 (0.6)	2.9 (0.8)	0.154
55 to 64	13.2 (1.5)	11.4 (2.5)	11.9 (1.9)	8.9 (1.6)	8.2 (1.1)	9.2 (1.0)	7.0 (0.8)	0.001
65+	18.3 (1.3)	21.6 (1.9)	23.9 (2.2)	20.4 (1.2)	19.2 (1.4)	16.3 (1.0)	17.9 (1.1)	0.022
Gender								
Men	7.3 (0.8)	6.8 (0.7)	7.8 (1.0)	7.1 (0.6)	7.0 (0.5)	7.3 (0.6)	6.5 (0.6)	0.529
Women	4.5 (0.5)	5.0 (0.6)	5.8 (0.7)	5.1 (0.6)	4.3 (0.3)	3.9 (0.4)	4.4 (0.4)	0.105
Race or ethnicity								
Whites	6.7 (0.4)	6.7 (0.6)	7.7 (0.8)	6.9 (0.6)	6.1 (0.5)	6.4 (0.5)	6.2 (0.6)	0.177
African Americans	4.0 (0.6)	5.8 (0.9)	4.7 (0.6)	5.9 (0.7)	4.4 (0.7)	4.7 (0.7)	4.4 (0.4)	0.668
Mexican Americans	2.6 (0.3)	2.6 (0.5)	2.8 (0.6)	3.1 (0.4)	3.1 (0.4)	3.7 (0.7)	2.6 (0.8)	0.414
Other	4.3 (0.5)	2.8 (0.7)	5.4 (1.9)	2.3 (0.7)	5.4 (0.8)	3.1 (0.6)	4.1 (0.6)	0.991
Education								
<High school	8.2 (0.6)	10.3 (1.0)	10.8 (1.9)	10.6 (1.1)	8.4 (0.5)	8.3 (1.0)	8.0 (0.9)	0.144
High school graduate or equivalent	7.0 (0.7)	6.0 (0.8)	7.1 (1.2)	6.4 (1.1)	6.2 (0.7)	7.1 (0.8)	7.0 (1.3)	0.806
>High school	4.0 (0.5)	4.2 (0.4)	5.3 (0.5)	4.6 (0.4)	4.3 (0.3)	4.1 (0.4)	4.2 (0.5)	0.733
Diagnosed diabetes								
Yes	21.4 (3.2)	19.2 (3.0)	21.4 (3.0)	21.0 (1.8)	20.0 (1.7)	17.6 (1.9)	19.3 (1.9)	0.425
No	4.7 (0.4)	4.9 (0.4)	5.4 (0.5)	4.7 (0.4)	4.1 (0.3)	4.3 (0.3)	3.8 (0.4)	0.014
BMI, kg/m^2^								
<25	3.9 (0.3)	3.3 (0.5)	5.5 (0.8)	3.4 (0.5)	4.1 (0.4)	3.5 (0.6)	3.5 (0.6)	0.368
25 to <30	6.7 (0.7)	5.7 (0.8)	7.2 (0.8)	7.1 (0.7)	5.3 (0.5)	4.9 (0.5)	5.2 (0.7)	0.034
≥30	7.2 (0.7)	8.0 (1.0)	8.0 (0.9)	7.2 (0.6)	7.0 (0.8)	8.0 (0.6)	7.3 (0.5)	0.873
Age‐adjusted results								
Total	6.3 (0.4)	6.4 (0.5)	6.9 (0.6)	6.1 (0.3)	5.5 (0.3)	5.3 (0.3)	5.2 (0.3)	0.001
Gender								
Men	8.4 (0.9)	7.9 (0.7)	8.4 (0.9)	7.7 (0.5)	7.4 (0.5)	7.4 (0.4)	6.6 (0.5)	0.043
Women	4.6 (0.5)	5.2 (0.5)	5.6 (0.7)	4.9 (0.6)	4.0 (0.3)	3.7 (0.4)	4.0 (0.3)	0.003
Race or ethnicity								
Whites	6.6 (0.4)	6.5 (0.6)	7.0 (0.7)	6.2 (0.4)	5.4 (0.4)	5.5 (0.4)	5.2 (0.4)	0.001
African Americans	5.4 (0.9)	7.5 (0.9)	5.6 (0.7)	7.1 (0.7)	4.9 (0.7)	4.9 (0.5)	4.8 (0.4)	0.037
Mexican Americans	4.6 (0.5)	5.5 (0.7)	5.4 (0.5)	4.4 (0.4)	5.2 (0.6)	5.6 (0.7)	4.8 (1.4)	0.908
Other	5.5 (0.9)	4.3 (0.8)	7.3 (2.5)	2.9 (0.8)	6.1 (0.7)	4.2 (0.8)	4.9 (0.7)	0.481
Education								
<High school	7.3 (0.7)	9.1 (1.0)	8.8 (1.3)	9.1 (0.9)	7.4 (0.6)	7.0 (0.8)	6.3 (0.7)	0.040
High school graduate or equivalent	7.2 (0.7)	6.3 (0.9)	6.8 (1.0)	5.6 (0.6)	5.7 (0.5)	6.5 (0.8)	6.1 (1.2)	0.404
>High school	5.5 (0.7)	5.4 (0.4)	6.5 (0.5)	5.5 (0.4)	4.8 (0.3)	4.3 (0.3)	4.5 (0.4)	0.008
Diagnosed diabetes								
Yes	14.3 (2.8)	13.6 (3.1)	13.1 (2.0)	12.0 (1.3)	12.2 (1.4)	9.6 (1.2)	11.6 (1.6)	0.150
No	5.6 (0.4)	5.7 (0.4)	6.0 (0.5)	5.2 (0.4)	4.5 (0.3)	4.6 (0.3)	4.1 (0.3)	<0.001
BMI, kg/m^2^								
<25	5.1 (0.4)	4.5 (0.6)	6.3 (0.8)	3.7 (0.5)	4.6 (0.4)	3.9 (0.5)	3.6 (0.4)	0.003
25 to <30	6.8 (0.7)	5.9 (0.7)	6.5 (0.7)	6.7 (0.5)	4.9 (0.4)	4.5 (0.4)	4.8 (0.5)	0.002
≥30	7.1 (0.8)	8.7 (0.9)	8.2 (0.9)	7.1 (0.4)	6.7 (0.8)	7.3 (0.5)	6.8 (0.4)	0.151

Based on data from the NHIS from 1980 to 1989, the age‐adjusted prevalence of self‐reported CHD among US adults aged 45 to 84 years varied between 2.2% and 2.6% with no clear trend.^44^ Recent data from the BRFSS showed that the prevalence of self‐reported CHD declined from 6.7% in 2006 to 6.0% in 2010 in adult populations aged ≥18 years.^45^ Declines were noted in all age groups, men and women, all education groups, and among whites and Hispanics but not among blacks, Asians or Native Hawaiians/Other Pacific Islanders, and American Indians or Alaska Natives.

In several NHANES, electrocardiograms (ECGs) were administered to adults aged 40 to 74 years. However, recent NHANES have not included this component. The percentages of adults with possible or probable ECG‐defined MI were 3.6% during 1971–1975, 3.4% during 1976–1980, and 2.4% during 1988–1994.^41^

Among successive groups of Framingham Study participants who were aged 55 to 64 years in 1953, 1963, and 1973, the prevalence of CHD among men increased from 10.2% in 1953 to 15.9% in 1973 (*P*=0.003) and that among women from 5.5% in 1953 to 6.9% in 1973 (*P*=0.250).^46^ CHD was defined as MI, coronary insufficiency, angina pectoris, and sudden and non‐sudden death from CHD.

Period prevalence of MI (hospitalization for MI or an out‐of‐hospital death due to MI) in the Pee Dee area of South Carolina decreased from 642 per 100 000 population in 1978 to 469 per 100 000 population in 1985.^26^ This overall trend reflected a significant decrease among white men, nonsignificant decreases among black men and women, and a nonsignificant increase among white women.

A series of autopsy studies from Olmsted County, Minnesota provide an interesting perspective on the trend in the prevalence of CHD. Among adults aged >30 years, the prevalence of “significant coronary disease” increased from 23% during 1950–1954 to 51% during 1975–1979 and the prevalence of a MI scar ranged between 36% and 41%.^47^ A subsequent autopsy study showed that the prevalence of atherosclerosis declined among adults aged 20 to 59 years (1979–1983: 38%; 1984–1989: 36%; 1990–1994: 27%; *P* for trend=0.02) but not adults aged ≥60 years (1979–1983: 61%; 1984–1989: 70%; 1990–1994: 59%; *P* for trend=0.44) from 1979 to 1994.^48^ A more recent autopsy study among residents aged 16 to 64 years from 1981 to 2004 showed declines in the prevalence of any coronary artery disease and mean grade.^49^

## Risk Factors

Impressive changes in major risk factors for CHD have occurred since the 1960s when national data about many of these risk factors first became available. The per capita cigarette consumption in the United States increased tremendously from 1900 into the 1960s. Subsequent to the first Surgeon General's Report in 1964, cigarette consumption started to decline and has reached levels last seen during the 1930s.^50^ In concert, the prevalence of smoking has decreased precipitously from 42.4% in 1965 to 19.3% in 2010.^51^ Furthermore, the exposure to second‐hand tobacco smoke has also declined.^52^

Concentrations of total cholesterol, non‐high‐density lipoprotein cholesterol, and low‐density lipoprotein cholesterol have decreased. Among adults aged 20 to 74 years, mean concentrations of total cholesterol were 222 mg/dL during 1960–1962, 216 mg/dL during 1971–1975, 215 mg/dL during 1976–1980, 204 mg/dL during 1988–1994, and 203 mg/dL during 1999–2002.^53^ Among adults aged ≥20 years, mean concentrations of total cholesterol were 206 mg/dL during 1988–1994, 203 mg/dL during 1999–2002, and 196 mg/dL during 2007–2010; mean concentrations of high‐density lipoprotein cholesterol were 50.7 mg/dL during 1988–1994, 51.3 mg/dL during 1999–2002, and 52.5 mg/dL during 2007–2010; mean concentrations of non‐high‐density lipoprotein cholesterol were 155 mg/dL during 1988–1994, 152 mg/dL during 1999–2002, and 144 mg/dL during 2007–2010; and mean concentrations of low‐density lipoprotein cholesterol were 129 mg/dL during 1988–1994, 123 mg/dL during 1999–2002, and 116 mg/dL during 2007–2010.^54^ In addition, control of hypercholesterolemia has also improved.^55^

The trend in hypertension has been more complicated.^56–58^ Among adults aged 18 to 74 years, the age‐adjusted prevalence of hypertension was 29.7% during 1960–1962, 36.3% during 1971–1974, 31.8% during 1976–1980.^56^ Among adults aged ≥20 years, the age‐adjusted prevalence of hypertension was 29.6% during 1999–2000, 29.0% during 2001–2002, 30.7% during 2003–2004, 29.9% during 2005–2006, 30.6% during 2007–2008, and 29.5% during 2009–2010.^58^ Both publications used a similar definition of hypertension (systolic blood pressure ≥140 mm Hg, diastolic blood pressure ≥90 mm Hg, or use of antihypertensive medication). Thus, the prevalence of hypertension has shown little change since 1988–1994. However, control of hypertension is improving.^57–59^ Of adults with hypertension, 33.2% were controlled during 1999–2002 compared with 45.8% during 2005–2008.^59^

National data sets provide few insights about the long‐term changes in physical activity. Analyses of data from the NHIS show that 14.3% of adults aged ≥18 years in 1998, 15.0% in 2000, 19.1% in 2009, and 20.7% in 2010 met the 2008 Physical Activity Guidelines for Americans (both aerobic activity [≥150 minutes/week of moderate‐intensity, 75 minutes/week of vigorous‐intensity aerobic physical activity, or an equivalent combination of moderate‐and vigorous‐intensity aerobic activity] and muscle‐ strengthening activities [≥2 days/week of muscle‐strengthening activities involving all major muscle groups of moderate or high intensity]).^60^ This apparent increase in leisure‐time physical activity may have been counterbalanced by unfavorable trends in energy expenditure at work and sedentary behavior. From 1960–1962 to 2003–2006, estimated mean daily energy expenditure at work among men and women declined by more than 100 calories.^61^ Sedentary behavior as exemplified by screen time (the amount of time that people spend watching television and videos, playing video games, or using a computer) has increased nationally.^62^

Weight and height have been measured in national surveys in the United States since 1960–1962. Among adults aged 20 to 74 years, the prevalence of obesity (body mass index ≥30 kg/m^2^) was 13.4% during 1960–1962, 14.5% during 1971–1974, 15.0% during 1976–1980, 23.3% during 1988–1994, and 30.9% during 1999–2000.^63^ Among adults aged ≥20 years, the prevalence of obesity (body mass index ≥30 kg/m^2^) was 30.5% during 1999–2000, 30.6% during 2001–2002, 32.2% during 2003–2004, 34.3% during 2005–2006, and 33.8% during 2007–2008, and 35.7% during 2009–2010.^64–65^ Abdominal obesity has also increased since 1988–1994.^66–67^

In the wake of the stark rise in obesity, the prevalence of diabetes has increased substantially since 1976–1980. Using 1985 WHO criteria, the prevalence of diabetes among adults aged 40 to 74 years was 11.4% during 1976–1980 and 14.3% during 1988–1994.^68^ Using 2008 ADA criteria, the prevalence of diabetes was 15.3% during 1988–1994 and 17.5% during 2005–2006.^69^

## Predicted CHD Risk

Starting with the Framingham Risk Score,^70^ multiple CHD risk equations have been developed to estimate the risk of developing incident CHD over a defined period, generally 10 years. Because these risk equations integrate the effects of key risk factors for CHD, trends in the predicted risk for CHD may correlate with trends in incident CHD. Using risk equations contained in the Adult Treatment Panel III report, little change in predicted 10‐year risk for CHD was observed from the period 1988–1994 to 1999–2002 among US adults.^71^ During 1988–1994, 76.5% of adults had a predicted 10‐year risk of <10%, 11.2% had a predicted 10‐year risk of 10% to 20%, and 12.3% had a predicted 10‐year risk of >20%. During 1999–2002, these percentages were 75.6%, 11.4%, and 13.0%, respectively. A subsequent analysis of national data showed that mean predicted 10‐year risk calculated using the Framingham Risk Score for CHD decreased from 10.0% during 1976–1980 to 7.9% during 1988‐1994 (*P*<0.001) and decreased from 7.9% during 1988–1994 to 7.4% during 1999‐2004 (*P*<0.001).^72^ The results from the latter study support the thesis of a decline in the incidence of CHD. A more recent analysis of NHANES data showed a continuing decline in predicted 10‐year risk from 1999–2000 to 2009–2010.^73^

### Incidence

Because incident CHD can manifest itself in different clinical presentations, measuring incident CHD can be challenging. A person may experience the first signs of CHD as angina pectoris and be treated on an outpatient basis. Another person may experience an MI as the first sign of CHD and be hospitalized. Someone else may die of sudden cardiac death. Thus, providing an integrated picture of all these possible first occurrences of CHD would require a system that is able to capture the spectrum of disease expression. However, such a system does not currently exist at the national level. Because national data about incident CHD are not available, our current knowledge of the true incidence of CHD in the United States comes from an amalgam of community surveillance (Table 4), cohort studies (Table 5), and health care delivery systems. Each of these sources of information has, to a variable degree, limitations that may include time frames, geographic coverage, and generalizability of the study populations.

**Table 4.** Community Studies of Incident Coronary Heart Disease or Sudden Death in the United States

Reference	Study	CHD Event	Period	Group	Rates or Percent Change
Elveback^7,74^	Rochester, Minnesota	Incident CHD (angina, MI, sudden unexpected death): medical, hospital, and autopsy records	1950–1954 to 1955–1959 to 1979–1982	Total	589 to 699 to 559/100 000 population*
Gillum^23^	Minnesota Heart Survey	Sudden death: death certificates	1970–1978	Men	311 to 244/100 000 population*
				Women	96 to 70/100 000 population*
Goldberg^25^	Worcester Heart Attack Study	MI. Review of medical records: history, enzymes, ECG. Autopsy records	1975–1984	Total	255 to 186/100 000 population*
Goldberg^75^	Worcester Heart Attack Study	MI. Review of medical records: history, enzymes, ECG. Autopsy records	1975–1988	Men	323 to 240/100 000 population*
				Women	176 to 137/100 000 population*
McGovern^18^	Minnesota Heart Survey	Acute CHD: ICD‐9 410‐411. Hospital records were abstracted; computer‐based algorithm	1985 to 1990	Men	315 to 298/100 000 population*
				Women	111 to 107/100 000 population*
Goff^76^	Corpus Christi Heart Project	MI hospitalizations. Review of medical records: ECG, enzymes, cardiac pain	1988–1989 to 1991–1992	Mexican American women	353.5 to 205.3/100 000 population*
				Non‐Hispanic White women	224.3 to 150.0/100 000 population*
				Mexican American men	485.8 to 367.4/100 000 population*
				Non‐Hispanic White men	345.9 to 342.2/100 000 population*
Rosamond^11^	Atherosclerosis Risk in Communities Study	MI hospitalizations. Hospital records were abstracted (symptoms, history, enzymes, ECG); computer‐based algorithm	1987–1994	Women	1.9 to 1.8/1000 persons*
				Men	4.1 to 4.1/1000 persons*
Cobb^77^	Seattle, Washington	Cardiac arrests receiving advanced life support. Medical incident reports supplemented with information from death certificates and hospital admissions	1979–1980 to 1999–2000	Total	1.39 to 0.91/1000 population*
				Men	2.15 to 1.24/1000 population*
				Women	0.68 to 0.61/1000 population*
		Cardiac arrest with ventricular fibrillation as first recorded rhythm		Total	0.85 to 0.38/1000 population*
				Men	1.39 to 0.60/1000 population*
				Women	0.35 to 0.17/1000 population*
Polentini^78^	Milwaukee, Wisconsin	Emergency medical services database	1992–2002	Total	37.1 to 19.4/100 000 population*
Floyd^32^	Worcester Heart Attack Study	MI. Review of medical records: history, enzymes, ECG	1975 to 1981 to 2005	Total	277 to 320 to 209/100 000 population*
Roger^34^	Olmsted County, Minnesota	MI. Review of medical records: cardiac pain, biomarkers (CK, CK‐MB, troponin), ECG. Computer‐based algorithm	1987–2006	All MI	186 to 180/100 000 population*
				CK/CK‐MB MI	186 to 141/100 000 population*
McManus^35^	Worcester Heart Attack Study	MI hospitalizations. Review of medical records: history, enzymes, ECG	1997–2005	STEMI	121 to 77/100 000 population*
				NSTEMI	126 to 132/100 000 population*
Rosamond^38^	Atherosclerosis Risk in Communities Study	MI hospitalizations. Hospital records were abstracted: chest pain, biomarkers, ECG. Computer‐based algorithm	1987–2008	All MI	
				Men	3.8%/year ↓*
				Women	3.5%/year ↓*
				White men	4.3%/year ↓*
				White women	3.8%/year ↓*
				Black men	1.5%/year ↓*
				Black women	2.9%/year ↓*
				STEMI	
				Men	4.8%/year ↓*
				Women	4.1%/year ↓*
				White men	5.4%/year ↓*
				White women	4.4%/year ↓*
				Black men	2.2%/year ↓*
				Black women	3.3%/year ↓*
				NSTEMI	
				Men	4.3%/year ↓*
				Women	4.2%/year ↓*
				White men	4.8%/year ↓*
				White women	4.5%/year ↓*
				Black men	2.0%/year ↓*
				Black women	3.9%/year ↓*

ECG indicates electrocardiograms; MI, myocardial infarction; NSTEMI, non ST‐segment elevation myocardial infarction; STEMI, ST‐segment elevation myocardial infarction.

* Statistically significant change.

* Change was not statistically significant.

* Statistical significance of change was not reported.

**Table 5.** Cohort Studies Reporting on Incidence of Coronary Heart Disease in Selected Locations in the United States

Reference	Study	CHD Event	Period	No. of Incident Events	Sample Size, Gender	Age at Baseline	Group	Change in Rates or Percentage Change
Pell^24^	Du Pont Company	M; review of medical records, ECG	1957–1959 to 1981–1983	6286 MI	Men: 2 304 958 PY	25 to 64	Total	3.19 to 2.29 per 1000*
			1957–1963 to 1978–1983	150 MI	Women: 426 150 PY			0.37 to 0.32 per 1000*
Reed^9^	Honolulu Heart Program	CHD: Medical review of hospital discharge and mortality records	1966–1984	674 CHD, 327 CHD deaths	7681 men	45 to 68	Men	6.1 to 5.7/1000 PY*
D'Agostino^46^	Framingham Heart Study	CHD: Medical review of MI, angina, sudden and nonsudden death from CHD, coronary insufficiency	1953–1963, 1963–1973, 1973–1983	―	526, 535, 581 men	55 to 64	Men	187 to 208/1000*
					689, 782, 812 women		Women	131 to 110/1000*
Sytkowski^10^	Framingham Heart Study	CHD: Medical review of MI, angina, sudden and nonsudden coronary death, coronary insufficiency	1950–1960–1970	928 CHD	618, 586, 598 men	50 to 59	Men	354 to 346/1000*
					757, 816, 834 women		Women	218 to 175/1000*
Hu^79^	Nurses' Health Study	Nonfatal MI or fatal coronary disease. Review of medical records. Deaths from state vital records, National Death Index, reports by next of kin or postal system	1980–1982 to 1992–1994	946 nonfatal MI, 358 fatal CD	85 941 women	34 to 59	≤49 years	25 to 13/100 000 PY*
							50 to 54 years	103 to 53/100 000 PY*
							55 to 59 years	177 to 149/100 000 PY*
							Total	31% ↓*
Ergin^28^	National Health and Nutrition Examination Survey I EFS	CHD: Hospital and nursing home discharge records, death certificate records. No review	1971–1975 to 1982–1984, 1982–1984 to 1992	1501, 778 CHD	10 869 men, women (1971–1982), 9774 men, women (1982–1992)	35 to 74	Total	133.3 to 113.5/10 000 PY*
		MI: Hospital and nursing home discharge records, death certificate records. No review		583, 358 MI			Total	49.7 to 49.2/10 000 PY*
Parikh^31^	Framingham Heart Study, Framingham Heart Study Offspring	MI‐ECG: Ischemic chest discomfort with diagnostic ECG changes, ±biomarker changes	1960s–1990s	639 MI‐ECG	9824 men, women	40 to 89	Total	≈50% ↓*
		MI‐biomarker: Ischemic chest discomfort with diagnostic biomarkers but no ECG changes		302 MI‐biomarker			Total	≈100% ↑*

ECG indicates electrocardiograms; MI, myocardial infarction.

* Statistically significant change.

* Change was not statistically significant.

* Statistical significance of change was not reported.

#### Community surveillance

One of the earliest studies to examine trends in the incidence of CHD emanated from Rochester, Minnesota.^7,74^ The age‐ and sex‐adjusted rates (per 100 000) of CHD incidence comprising angina pectoris, MI, and sudden unexpected death were 589 during 1950–1954, 699 during 1955–1959, 589 during 1960–1964, 571 during 1965–1969, and 572 during 1970–1974, 538 during 1975–1978, and 559 during 1979–1982. The rate among men generally decreased, whereas the rate among women increased slightly. The age‐adjusted rate (per 100 000) of sudden unexpected death decreased from 126 during 1950–1954 to 73 during 1979–1982. Rates of angina pectoris decreased from 240 to 213, whereas rates of MI increased from 222 to 255 during the same period.

A more recent study from Olmsted County, Minnesota showed that the age‐ and sex‐adjusted rate (per 100 000) of hospitalizations for incident MI from 1987 to 2006 changed from 186 to 180 (*P*=0.171).^34^ When MI hospitalizations were restricted to those that used creatine kinase/creatine kinase‐MB but not troponin for the diagnosis of MI, a significant decrease in the rate from 186 to 141 was observed. Furthermore, rates of STEMI declined significantly by 41% when troponin was considered or 44% when troponin was excluded. However, rates of incident NSTEMI increased by 49%. An interesting aspect of this study is that measurements of creatine kinase/creatine kinase‐MB continued to be administered from 2000 on as troponin was being ushered in, thus allowing an evaluation of the impact of changing diagnostic criteria on trends in MI incidence.

From 1970 to 1978, out‐of‐hospital mortality rates from CHD in Minneapolis and St. Paul, Minnesota declined by 43% among men and by 40% among women.^23^ Another study in Minneapolis and St. Paul found that the age‐adjusted hospitalization rates for first MI declined by 5% among men and 4% among women from 1985 to 1990 based on ICD‐9‐CM codes of 410 and 411 obtained from 31 hospitals in 1985 and 25 hospitals in 1990 among patients aged 30 to 74 years.^18^ These changes were not statistically significant.

From 1988 to 1992, the age‐adjusted incidence rates of hospitalized MI in the Corpus Christi Heart Project decreased significantly among Mexican‐American women.^76^ Nonsignificant reductions were reported for white women and Mexican‐American men, and little change was reported for white men.

In Seattle, the age‐ and sex‐adjusted incidence rates of cardiac arrest with ventricular fibrillation from 1980 to 2000 declined by 56%, and the incidence of all treated arrests declined by 34%.^77^ Declines in the incidence rates of cardiac arrest with ventricular fibrillation were similar for men and women, but the decline in the incidence rates of all treated arrests in men exceeded that in women.

As part of the ARIC study, surveillance of hospital admissions for MI among residents aged 35 to 74 years was conducted among adults aged 35‐74 years in 4 communities (Forsyth County, NC; Jackson, MS; Minneapolis suburbs, MN; Washington County, MD) from 1987 to 1994.^11^ Hospital discharges meeting certain ICD‐9‐CM codes from 28 hospitals were reviewed, and a computerized algorithm assigned a diagnosis using information on symptoms, cardiac enzymes, and ECGs collected by study personnel from medical records. A total of 11 869 hospitalizations for MI were estimated. The age‐adjusted rate of hospitalizations for incident MI in women was 1.9 per 1000 population in 1987 and 1.8 per 1000 population in 1994, whereas the rate in men remained unchanged at 4.1 per 1000 population. The average annual rate of change during the study period was +2.9% among black men, +7.4% among black women, −2.5% among white women, and −0.3% among white men. Out‐of‐hospital mortality attributed to CHD declined by 3.6% per year.

More recently, updated results of this surveillance system in these 4 communities from 1987 to 2008 showed that the age‐adjusted rate of hospitalizations for incident MI declined.^38^ The basic surveillance methodology remained largely the same. Because this study covered a period that saw profound changes in the use of diagnostic biomarkers (the advent of troponin), the study authors made a number of adjustments in their analytic strategy to account for these changes. For this study, 30 985 hospitalizations for MI were estimated. The age‐ and biomarker adjusted rate of hospitalization for a first MI changed by −4.3% (95% CI: −4.7, −3.8) among white men, −3.8% (95% CI: −4.5, −3.1) among white women, −1.5% (95% CI: −2.7, −0.4) among black men, and −2.9% (95% CI: −4.2, −1.5) among black women. Compared to the period 1987–1996, the decline in the rates of combined hospitalization for incident MI or fatal CHD during 1997–2008 accelerated. Declines were observed in the age‐ and biomarker‐adjusted rate of hospitalization for both STEMI and NSTEMI. The authors noted that the patterns in rates based only on ECG criteria and clinical history mirrored rates that included biomarker data. Out‐of‐hospital mortality attributed to CHD declined by 5.6% per year among white men, 4.4% per year among white women, 2.7% among black men, and 2.6% among black women. Declines in both sexes during the period 1997–2008 far exceeded the declines during the period from 1987 to 1996.

Surveillance of MI among residents of Worcester, Massachusetts as part of the Worcester Heart Attack Study has been conducted since 1975.^25,27,32,75^ Hospitalizations for MI were identified, and medical records for these hospitalizations were reviewed. Information about the clinical history, ECG changes, and biomarker changes was abstracted to make a determination of MI. Although the age‐adjusted hospitalization rates (per 100 000) for incident MI dropped from 277 in 1975 to 209 in 2005, the rates during intervening years varied considerably.^32^ A subsequent investigation of trends in incident hospitalizations for MI from 1997 to 2005 showed that the incidence rate for STEMI was 121 in 1997, peaked in 1999 and then declined progressively to 77 through 2005.^35^ In contrast, the incidence rate of NSTEMI spiked in 2001 and then declined reaching a level in 2005 (132) that was similar to that in 1997 (126). From 1975 to 1988, out‐of‐hospital mortality rates attributed to CHD declined by 60% among men and 69% among women.^75^

In Milwaukee, the incidence (per 100 000) of treated cardiac arrest with ventricular fibrillation or tachycardia as the first recorded rhythm declined from 37.1 in 1992 to 19.4 in 2002.^78^ The incidence of all treated arrests was 82.8 in 1992 and 82.3 in 2002.

These community surveillance studies provide strong evidence that incidence has decreased in those areas although the onset of the decline varied by geographical location with the earliest decline being observed in Olmsted County, Minnesota. These well‐conducted studies employed standardized case‐definitions for MI. Two of these studies also carefully navigated the changing currents in diagnostic criteria for MI. Nevertheless, a drawback of these studies remains their narrow geographic focus.

#### Cohort studies

A study of male employees of Du Pont Company showed that the age‐adjusted incidence rate (per 1000) of first MI decreased steadily from 3.19 during 1957–1959 to 2.29 during 1981–1983.^24^ Events were identified from insurance claims and death certificates, and medical records were reviewed.

In a cohort of 8006 men of Japanese ancestry living on Oahu, the incidence of CHD increased from 1966 to 1978 and then decreased through 1984.^9^ For the entire study period, the estimated annual change in incidence was −0.4% (95% CI: −2.6%, +1.8%). Incident CHD events included CHD deaths (ICD‐8 codes 410‐414 as the underlying or contributing cause of death or sudden unexplained deaths within 1 hour of being well) and nonfatal MI (ECG evidence and/or cardiac enzyme changes).

An early analysis of data from the Framingham Heart Study found that the incidence of CHD among 3 successive cohorts of men and women aged 55‐64 years did not change significantly from 1953–1963 to 1973–1983.^46^ For men, incidence rates (per 1,000) for CHD were 187 during 1953–1963, 210 during 1963–1973, and 208 during 1973–1983 (*P* trend=0.41), and incidence rates for MI were 103 during 1953–1963, 116 during 1963–1973, and 120 during 1973–1983 (*P* trend=0.42). For women, incidence rates for CHD were 131 during 1953–1963, 132 during 1963–1973, and 110 during 1973–1983 (*P* trend=0.41), and incidence rates for MI were 38 during 1953–1963, 50 during 1963–1973, and 45 during 1973–1983 (*P* trend=0.38). CHD was defined as MI, coronary insufficiency, angina pectoris, and sudden and non‐sudden death from CHD. MI was determined from serial ECG changes and cardiac enzymes when they became available.

In a subsequent analysis of data from the Framingham Heart Study, 20‐year incidence of CHD in 3 consecutive cohorts of adults aged 50 to 59 years was examined.^10^ CHD included MI, angina, sudden and non‐sudden coronary death, and coronary insufficiency. Among women, the incidence (per 1000) of CHD decreased significantly from 218 events in the 1950 cohort to 175 events in the 1970 cohort (*P*<0.05). In contrast, the rate among men was 354 in the 1950 cohort and 346 in the 1970 cohort.

Another analysis of data from the Framingham Heart Study and Framingham Heart Study Offspring Cohort found that the risk of sudden coronary death in adults without CHD or congestive heart failure decreased by 39% from 1950–1969 to 1990–1999.^80^

More recently, data from the Framingham Heart Study and Framingham Heart Study Offspring Cohort showed that the incidence of MI had declined during successive decades starting with 1960–1969 and ending with 1990–1999.^31^ Among 9824 participants aged 40 to 89 years, 941 MIs were recorded of which 639 were defined on the basis of ECG changes and 302 on the basis of biomarker changes. MIs were identified by information obtained from study participants during follow‐up examinations or mailings of update questionnaires and evaluated with medical records. MIs were divided into 2 groups: those with ischemic chest discomfort and diagnostic ECG changes irrespective of diagnostic biomarker changes (MI‐ECG) and those with ischemic chest discomfort and diagnostic biomarker changes without diagnostic ECG changes (MI‐marker). Rates of incident MI‐ECG dropped by about half, whereas rates of incident MI‐marker doubled. Significant decreases in MI‐ECG were noted for men aged 50 to 59 years and 70 to 79 years as well as women aged 70 to 79 years. In contrast, significant increases in ECG‐marker were noted for men aged 50 to 59 years and 70 to 79 years as well as women aged 70 to 79 years. The authors concluded that much of the uncertainty in trends in the incidence of MI may have been attributable to changes in diagnostic criteria for MI.

Data from the Nurses' Health Study that included 85 941 participants aged 34 to 59 years showed that the incidence of CHD declined by 31% from 1980–1982 to 1992–1994.^79^ CHD was defined as nonfatal MI or fatal coronary disease. The former was determined from medical record review of MIs reported by the study participants, and MI was defined using World Health Organization criteria. Deaths were determined from state vital records, the National Death Index, reports by next of kin, and the postal system. In all, 946 participants had a nonfatal MI and 358 experienced death attributable to coronary disease.

An analysis of data from the National Health and Nutrition Examination Survey I Epidemiologic Follow‐up Study from 1971 to 1992 showed that the age‐adjusted incidence (per 10 000) declined from 133.3 from 1971–1975 to 1982–1984 to 113.5 from 1982–1984 to 1992 for CHD and from 49.7 to 49.2, respectively, for MI.^28^ The incidence of CHD declined significantly among white men (−14.6%) and women (−11.4%). The relative decrease among black men (−18.5%) was the largest of the 4 groups but failed to reach statistical significance. The decrease among black women (−3.8%) was the smallest of any of the 4 groups. The baseline cohort included 10 869 participants aged 35 to 74 years, and the follow‐up cohort included 9774 participants aged 35 to 74. Incident CHD was defined as a death from CHD, a hospitalization for CHD, or a nursing home stay with the ICD‐9 codes of 410‐414. Prevalent CHD was excluded from the baseline cohort on the basis of self‐reported heart attack, heart failure, or stroke as well as the use of medications used to treat heart disease.

The cohort studies provide valuable insights into trends of incident CHD in their study populations, which range from relatively specific populations such as employees of a company to near representative samples of US adults. Thus, generalizability of their findings to the national level is a prime limitation. Also, the age range of participants of many cohort studies is limited precluding an examination of trends in incidence across the full adult lifespan. By examining the experience of the participants who have been repeatedly examined, a Hawthorne‐type of effect could be introduced into studies in that study participants may alter their behaviors in response to their study participation. On the other hand, cohort studies often use some of the best‐validated measures of incident CHD and yield information over some of the longest time frames.

#### Health care delivery systems

The large health care delivery systems potentially represent an important opportunity for conducting surveillance of CHD in large segments of the US population. Drawing from the administrative systems of Kaiser Permanente Northern California, investigators identified hospitalizations for MI from 1999 to 2008 using the ICD‐9‐CM code of 410 and divided these into hospitalizations for STEMI (ICD‐9‐CM codes 410.0‐410.6, 410.8) and NSTEMI (410.7, 410.9).^33^ A total of 46 086 hospitalizations for incident MI among adults aged ≥30 years were included in the analyses. The age‐ and sex‐adjusted rate (per 100 000 person‐years) of hospitalizations for incident MI were 274 in 1999 and 287 in 2000 and then progressively declined to 208 through 2008. Rates of STEMI decreased steadily throughout the study period from 133 to 50, but the rates of NSTEMI increased until 2004 and began to decrease in subsequent years. Validation studies were performed to show that the positive predictive value for the STEMI and NSTEMI coding algorithm did not materially change during the study period.

Because health care delivery systems represent an important source of health care and coverage, the databases and expanding rich electronic medical records of these health systems contain potentially valuable information about trends in the incidence of CHD of their memberships. However, information from these data systems is subject to several considerations: data from these plans generally may not reach back far in time, the need to validate electronic data sources deserves careful consideration, and health care delivery systems may not be fully representative of all relevant populations (eg, uninsured persons).

#### Population surveys

Data from the National Health Interview Survey have been analyzed to examine trends in the incidence of CHD.^44^ Participants who reported that they had CHD, angina pectoris, myocardial infarction, or heart attack with an onset during the 12 months prior to their interview were considered to have had an incident event. From 1980 to 1989, the age‐adjusted incidence per 1000 population ranged between 2.2 and 3.2 with no significant linear trend. Among white men, a nonlinear trend was described with decreasing rates from 1986 to 1989. Among white women, a significant increase in the incidence rate was observed.

## Unrecognized MI

Some percentage of MIs are not diagnosed because patients are asymptomatic, experience symptoms that do not prompt them to seek medical care, or experience symptoms that may be insufficiently characteristic of an MI and do not result in a diagnostic evaluation.^81^ Thus, these MIs are typically recognized when patients receive an ECG examination subsequent to the MI. Such MIs are also referred to as silent, asymptomatic, or undiagnosed MIs. The prevalence of unrecognized MIs has been reported to range from 4.3% to 44%,^81–82^ and factors like the age and gender distribution of study participants account in part for the wide range in estimates. Little about possible trends in unrecognized MI is known, and the impact of this category of MI on the trends in incidence of MI is unclear. Despite clinical impressions that persons with diabetes experience more painless MIs, it remains uncertain whether the increased prevalence of diabetes may have affected trends in unrecognized MI because diabetes has not been shown to be an independent predictor of unrecognized MI.^81^ For cohort studies such as the Framingham Heart Study that administer periodic ECGs, unrecognized MIs should have been captured, and the trends in incident CHD reported from such cohort studies should not be biased by the exclusion of unrecognized MIs, although Framingham included a selected patient sample that may not be representative of the broader US population. The results from other studies that rely on identifying patients with MI who present for medical care could be biased depending on the direction and strength of the trends in unrecognized MI.

## Severity of MI

Successful primary prevention that reduces the incidence of CHD may favorably shift the distribution of severity of MI. Consequently, evidence of a change in the severity of MI may provide indirect support for a reduction in the incidence of CHD. Data from several studies suggest that the severity of MIs has lessened.^34–35,34–88^ Initial reports from Worcester, Massachusetts found that the incidence of cardiogenic shock complicating an MI did not change significantly from 1975 to 1988 or from 1975 to 1997.^83–84^ A more recent report noted that there was evidence of a decline in cardiogenic shock from the late 1990s to 2005.^87^ A decline in the incidence of STEMI but not NSTEMI in Worcester also suggests that the severity of MI declined in that area.^35^ An initial report from ARIC investigators yielded inconsistent evidence that the severity of MIs had decreased from 1987 to 1994.^85^ However, a subsequent report covering the period from 1987 to 2002 noted that the severity of MI had declined.^88^ An investigation conducted in Olmsted County, Minnesota showed that the severity of MI had decreased from 1983 to 1994.^86^ A more recent study from 1987 to 2006 noted declines in the proportion of MIs with Killip class 2‐4 and with ST‐segment elevation.^34^ Furthermore, the decline in hospitalizations for incident STEMI not paralleled by a similar decline in NSTEMI in the Kaiser Permanente Northern California system argues for a decline in the severity of MI.^33^ Because severity of an MI reflects a complex mix of pathophysiologic underpinnings, patient behavior in seeking medical care, comorbidities, and medical care, studying temporal trends in severity is a complicated endeavor.^89–90^ Nevertheless, the available evidence suggests some degree of concordance between improving trends in MI severity and incident CHD.

## Summary and Closing Thoughts

Although a complete picture of the national trend in CHD incidence in the United States remains elusive, the findings from community‐based studies, prospective studies, and health care delivery systems reporting decreases in incidence of CHD provide the most convincing evidence that the national incidence of CHD may have declined. These findings are buttressed by data showing declines in national rates of death attributed to CHD, studies showing decreases in sudden death and out‐of hospital mortality associated with MI, declines in hospitalizations for CHD, improving MI severity, possible recent declines in prevalence of CHD, declines in predicted 10‐year risk, and favorable improvements in the prevalence and control of some major CHD risk factors. Although each of these pieces of information is an imperfect reflection of incident CHD, in the aggregate they tell an increasingly compelling story of the evolution of CHD incidence in the United States. Declines in death from CHD are potentially suggestive of declining CHD incidence if the declines in case‐fatality rates do not account for the entire decrease in mortality.

Because the studies examining trends in CHD incidence covered different time frames and were conducted in different areas of the United States, pinpointing the exact time when incidence started to decrease is difficult because the onset of the start of any declines may have varied geographically. Community surveillance studies have reported decreases in incidence as early as the 1960s (Rochester, MN),^7^ during the late 1980s (Corpus Christi Heart Project),^76^ 1990s (ARIC),^38^ and 2000s (Worcester Heart Attack Study).^32^ Other studies suggest that decreases in incidence occurred during the 1960s (Framingham Heart Study, the Du Pont Company),^10,24^ 1970s (Framingham Heart Study),^31^ 1980s (Nurses' Health Study, NHANES Epidemiologic Follow‐up Study, Framingham Heart Study),^28,31,79^ 1990s (Framingham Heart Study, Nurses' Health Study),^31,79^ and 2000s (Kaiser Permanente Northern California).^33^

Three of the studies illustrate the difficulty in interpreting surveillance data over long periods of time particularly when changes in diagnostic criteria occur.^31,34,38^ The introduction of troponin testing around the turn of the century marked an important change in the diagnostic criteria for MI^91^ and coincided with a shift in the ratio of STEMI to NSTEMI with decreases in rates of STEMI and increases in rates of NSTEMI. Furthermore, the advent of electron‐beam computed tomography and multi‐detector computed tomography to detect calcium in the walls of coronary arteries has led to earlier identification of CHD.^92^ From a surveillance point of view, these disruptive changes in diagnostic criteria emphasize the importance of being able to disentangle the effects on such changes on trend analyses.

Validation of incident CHD events enhances the credibility of trends in CHD incidence. The majority of community surveillance, cohort, and health care delivery system‐based studies included reviews of medical records searching for clinical presentation, electrographic criteria, and cardiac biomarkers to confirm the presence of CHD, although these validation efforts differed across studies and across time periods as diagnostic criteria were also evolving.

Furthermore, observational studies suggest that an enormous amount of CHD can yet be prevented by adopting healthy behaviors or by optimizing behavioral and clinical risk factors as exemplified by the AHA's 7 cardiovascular health metrics.^93–99^ In addition, initiatives such as the Million Hearts Initiative, which aims to prevent 1 million heart attacks and strokes by 2017 through a combination of clinical and community actions, will, if successful, potentially hasten the decline in the incidence of CHD.^100–101^

The data sources opening a window into race or ethnicity‐specific trends of CHD incidence are few. Data from the ARIC study suggest that African‐American men and women did enjoy declining CHD incidence, but the decline among African Americans manifested itself later than among whites and the size of the decline was smaller than that of whites. These results are corroborated by Medicare data and data from the NIS also showing that the hospitalization rate for MI declined more slowly among African Americans than among whites.^21–22^ Gaps in evidence exist about the trends in CHD incidence among other racial or ethnic groups such as Hispanics and Asians. Given the rapidly evolving demographic composition of the US population, data collection efforts to shed light on the evolution of CHD in major and growing racial and ethnic groups are needed. Perhaps, large health care delivery systems and growing health system‐based networks are best suited to provide such results if their expanding electronic medical record and other data systems capture valid racial and ethnic designations and relevant clinical outcomes of their memberships.

Efforts to establish community surveillance for CHD harken back decades.^102–103^ A national system to monitor CHD incidence has never been established, however, and this gap has not gone unnoticed.^104–107^ As part of its recommendations, the Institute of Medicine highlighted the critical importance of having data on the incidence of CVD and the need for a system that would collect such data. The report cited potential avenues such as the establishment of registries, the use of cohort studies, and the use of claims and electronic medical record data to accomplish such a goal. The development of a national system to monitor the trend in the incidence of CHD would help to fill this current void in the knowledge base of the epidemiology of CHD and provide critical data to improve cardiovascular health of the US population.

In conclusion, definitive data about national trends of incident CHD in the United States currently are not available, and, therefore, clues about these trends must be gleaned from a variety of auxiliary data sources. Studies in different parts of the country demonstrate improvements in the incidence of CHD that may have commenced several decades ago in some parts of the country, and an increasing number of recent studies have described favorable trends during the first decade of the 21st century. Taken together, these studies yield encouraging but tentative signals that the incidence of CHD in the United States may be waning. Bringing greater clarity to this important topic of cardiovascular epidemiology poses a pressing public health need.
